# Supplementary figures and images for: Increased Zygote-Derived Plantlet Formation through In Vitro Rescue of Immature Embryos of Highly Apomictic Opuntia ficus-indica (Cactaceae)
Source: Plants (Basel). 2023 Jul 25;12(15):2758. doi: 10.3390/plants12152758 (PMC10421068; doi:10.3390/plants12152758)

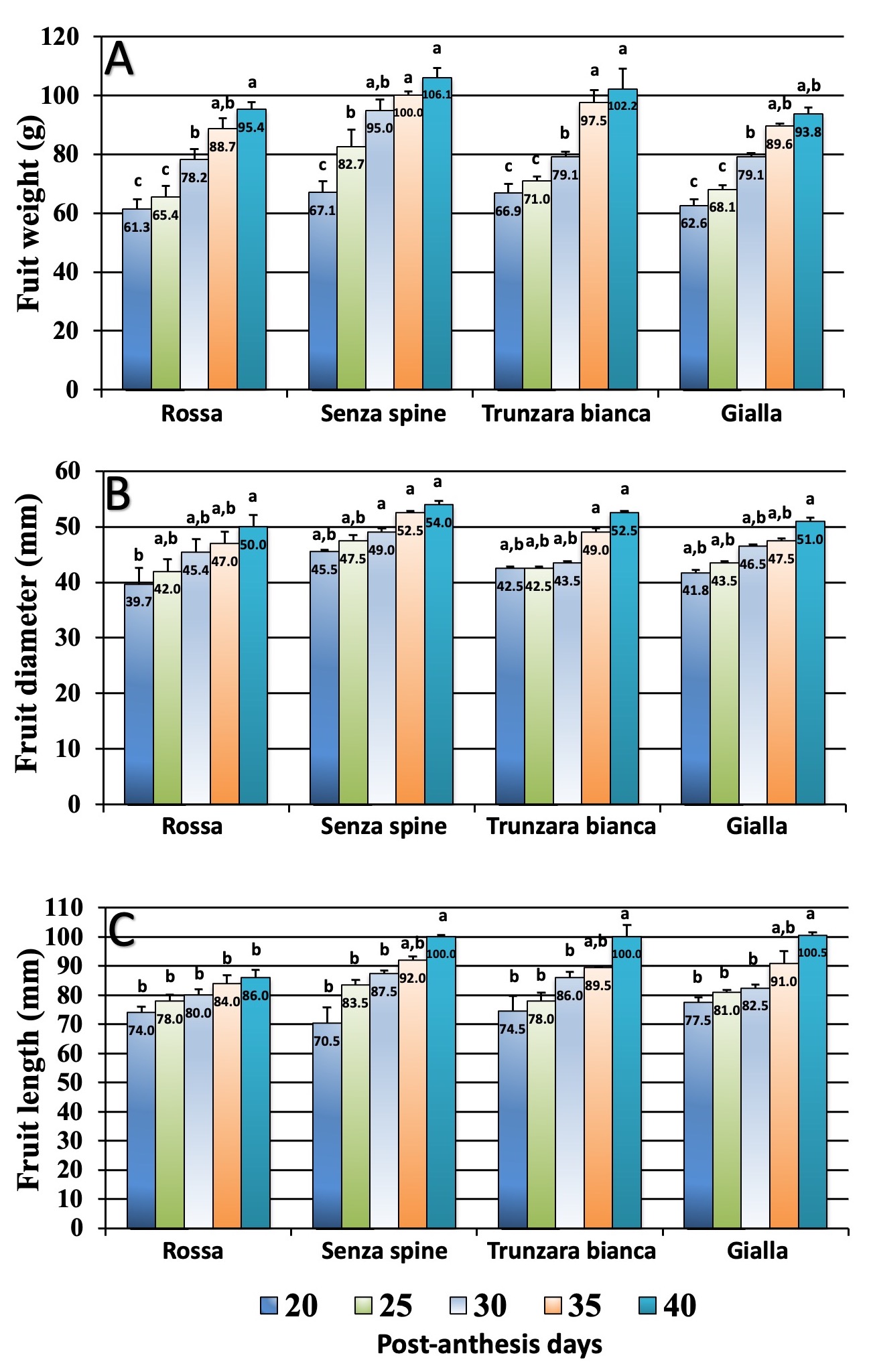

Supplement: Supplementary file 1 [file plants-12-02758-s001.zip › plants-2420089-supplementary-Figure S1.jpg]
